# Supplementary material for: HIVprotI: an integrated web based platform for prediction and design of HIV proteins inhibitors
Source: J Cheminform. 2018 Mar 9;10:12. doi: 10.1186/s13321-018-0266-y (PMC5845081; doi:10.1186/s13321-018-0266-y)
Supplement: Supplementary file 2 — Additional file 2. Source code of HIVProtI web server. [file 13321_2018_266_MOESM2_ESM.zip › HIVprotI_Source-code/hivprotI/weka-3-6-6/doc/weka/core/converters/FileSourcedConverter.html]

FileSourcedConverter


---


|  |  |  |
| --- | --- | --- |
| **Overview** \| **Package** \| **Class** \| **Tree** \| **Deprecated** \| **Index** \| **Help** \| **Weka's home** | |  |
| **PREV CLASS**   **NEXT CLASS** | **FRAMES**    **NO FRAMES**     **All Classes** |  |
| SUMMARY: NESTED \| FIELD \| CONSTR \| METHOD | DETAIL: FIELD \| CONSTR \| METHOD |  |


---


## weka.core.converters Interface FileSourcedConverter

**All Known Implementing Classes:**: AbstractFileLoader, AbstractFileSaver, ArffLoader, ArffSaver, C45Loader, C45Saver, CSVLoader, CSVSaver, LibSVMLoader, LibSVMSaver, SerializedInstancesLoader, SerializedInstancesSaver, SVMLightLoader, SVMLightSaver, XRFFLoader, XRFFSaver

---

``` public interface FileSourcedConverter ```

Interface to a loader/saver that loads/saves from a file source.

**Version:**
:   $Revision: 1.5 $

**Author:**
:   Mark Hall

---

| **Method Summary** | |
| --- | --- |
| `java.lang.String` | `getFileDescription()`             Get a one line description of the type of file |
| `java.lang.String` | `getFileExtension()`             Get the file extension used for this type of file |
| `java.lang.String[]` | `getFileExtensions()`             Gets all the file extensions used for this type of file |
| `boolean` | `getUseRelativePath()`             Gets whether relative paths are to be used |
| `java.io.File` | `retrieveFile()`             Return the current source file/ destination file |
| `void` | `setFile(java.io.File file)`             Set the file to load from/ to save in |
| `void` | `setUseRelativePath(boolean rp)`             Set whether to use relative rather than absolute paths |

**Method Detail**

### getFileExtension

```
java.lang.String getFileExtension()
```

:   Get the file extension used for this type of file

    :   **Returns:**: the file extension

---


### getFileExtensions

```
java.lang.String[] getFileExtensions()
```

:   Gets all the file extensions used for this type of file

    :   **Returns:**: the file extensions

---


### getFileDescription

```
java.lang.String getFileDescription()
```

:   Get a one line description of the type of file

    :   **Returns:**: a description of the file type

---


### setFile

```
void setFile(java.io.File file)
             throws java.io.IOException
```

:   Set the file to load from/ to save in

    :   **Parameters:**: `file` - the file to load from **Throws:**: `java.io.IOException` - if an error occurs

---


### retrieveFile

```
java.io.File retrieveFile()
```

:   Return the current source file/ destination file

    :   **Returns:**: a `File` value

---


### setUseRelativePath

```
void setUseRelativePath(boolean rp)
```

:   Set whether to use relative rather than absolute paths

    :   **Parameters:**: `rp` - true if relative paths are to be used

---


### getUseRelativePath

```
boolean getUseRelativePath()
```

:   Gets whether relative paths are to be used

    :   **Returns:**: true if relative paths are to be used


---


|  |  |  |
| --- | --- | --- |
| **Overview** \| **Package** \| **Class** \| **Tree** \| **Deprecated** \| **Index** \| **Help** \| **Weka's home** | |  |
| **PREV CLASS**   **NEXT CLASS** | **FRAMES**    **NO FRAMES**     **All Classes** |  |
| SUMMARY: NESTED \| FIELD \| CONSTR \| METHOD | DETAIL: FIELD \| CONSTR \| METHOD |  |


---
